# Melatonin Improves the Quality of *In Vitro* Produced (IVP) Bovine Embryos: Implications for Blastocyst Development, Cryotolerance, and Modifications of Relevant Gene Expression

**DOI:** 10.1371/journal.pone.0093641

**Published:** 2014-04-02

**Authors:** Feng Wang, XiuZhi Tian, YanHua Zhou, DunXian Tan, ShiEn Zhu, YunPing Dai, GuoShi Liu

**Affiliations:** 1 State Key Laboratory of Animal Nutrition, Key Laboratory of Animal Genetics and Breeding of the Ministry of Agriculture, National Engineering Laboratory for Animal Breeding, College of Animal Science and Technology, China Agricultural University, Beijing, China; 2 Department of Cellular & Structural Biology, The University of Texas Health Science Center, San Antonio, Texas, United States of America; 3 State Key Laboratories of Agro-biotechnology, College of Biological Sciences, China Agricultural University, Beijing, China; University of Connecticut, USA, United States of America

## Abstract

To evaluate the potential effects of melatonin on the kinetics of embryo development and quality of blastocyst during the process of *in vitro* bovine embryo culture. Bovine cumulus–oocyte complexes (COCs) were fertilized after *in vitro* maturation. The presumed zygotes were cultured in *in vitro* culture medium supplemented with or without 10^−7^ M melatonin. The cleavage rate, 8-cell rate and blastocyst rate were examined to identify the kinetics of embryo development. The hatched blastocyst rate, mortality rate after thawing and the relevant transcript abundance were measured to evaluate the quality of blastocyst. The results showed that melatonin significantly promoted the cleavage rate and 8-cell embryo yield of *in vitro* produced bovine embryo. In addition, significantly more blastocysts were observed by Day 7 of embryo culture at the presence of melatonin. These results indicated that melatonin accelerated the development of *in vitro* produced bovine embryos. Following vitrification at Day 7 of embryo culture, melatonin (10^−7^ M) significantly increased the hatched blastocyst rate from 24 h to 72 h and decreased the mortality rate from 48 h to 72 h after thawing. The presence of melatonin during the embryo culture resulted in a significant increase in the gene expressions of *DNMT3A*, *OCC*, *CDH1* and decrease in that of *AQP3* after thawing. In conclusion, melatonin not only promoted blastocyst yield and accelerated *in vitro* bovine embryo development, but also improved the quality of blastocysts which was indexed by an elevated cryotolerance and the up-regulated expressions of developmentally important genes.

## Introduction

Growing evidence indicates that for investigating early human embryo development, bovine embryo pre-implantation is superior to the mouse model [1]. However, numerous studies have shown that *in vitro*-produced bovine embryos differ from their *in vivo*-derived counterparts. These differences include timing of development, alterations of morphology [2], [3], metabolism [4], occurrence of mixoploidy [5], intact of zona pellucida [6], blastocyst numbers [7], tolerance of cryopreservation [8], relevant gene expression [9] and, ultimately, in pregnancy rates [10]. It is the intrinsic quality of the oocyte that determines the proportion of oocytes developing to blastocysts (i.e., oocyte developmental competence) while, the postfertilization culture environment has the biggest impact on blastocyst quality [11]–[13]. The culture conditions influence the timing of embryo development, such as the first cleavage division [14], an onset of major genomic activation of embryos at the eight- to 16-cell stage [15] and finally the blastocyst quality. For example, once the *in vitro*-produced bovine zygotes were implanted into the oviduct of ewe, this will dramatically increase their cryotolerance to a level similar to that of *in vivo*-produced embryos [16]. Rizos et al observed that even the postfertilization culture medium containing serum it would affect the processes of embryo development and the quality of the resulting blastocysts [17]. Therefore, the *in vitro* embryo culture environments determine the quality of embryos.

Melatonin (N-aceyl-5-methoxytryptamine) is a well known free radical scavenger, antioxidant, and anti-apoptotic agent [18]. It has been widely used in protecting against free radical damage in different animal models and this molecule has been suggested to have treatment utilizations in cancer, immunological disorders, Alzheimer's disease, diabetes and viral infections [19]–[22]. In recent years, melatonin has also been successfully used for promoting *in vitro* embryo development in mice [23], buffaloes [24], heifers [25] and sows [26], [27]. In mouse, the beneficial effects of melatonin on the development of fresh and vitrified 2-cell mouse embryos has been observed. These benefits include enhancement of the blastocyst formation rate, mean cell number/blastocyst and the rate of hatch ability [28]. Melatonin not only promoted the *in vitro* bovine embryo development under the high oxygen concentration, but also increased blastocyst rate of heat-stressed bovine oocytes in the *in vitro* conditions [25]. In other species, such as in porcine, melatonin (10^−9^ M) had a positive effect on embryo cleavage rates and blastocyst total cell numbers [26] and in ovine, melatonin improved *in vitro* embryonic quality and also their survival [29]. Recently, it was reported that a moderate level of melatonin (10^−6^ M) improved the development and hatchability rates of preimplantated rabbit embryos [30]. These investigations discussed above are mainly focus on the melatonin's effects on the morphological variations regarding the *in vitro* embryo development, such as cleavage rate, blastocyst rate, hatched blastocyst rate and blastocyst cell number [28], [31]. Therefore, it is necessary to further identify and evaluate the effects of melatonin on the quality of *in vitro* produced embryos. For example, our previous studies have shown that melatonin improved the quality of the *in vitro* development of mouse embryos reflected by the elevated efficiency of blastocyst implantation after embryo transferring [32].

Blastocyst mRNA expression and cryopreservability have been often used to serve as the sensitive indicators of embryo quality and developmental competence [17], [33], [34]. To our knowledge, little is known regarding the effects of melatonin on bovine blastocyst quality evaluated by blastocyst development, cryotolerance and the expression of the embryo-development relevant genes. Therefore, the current study will focus on the effects of melatonin on bovine embryo development and most importantly, its effects on the quality of *in vitro* produced embryos after thawing.

## Materials and Methods

### Chemicals and Solutions

NaCl, KCl, NaHCO3, Na pyruvate, hemicalcium L-lactate, BSA, L-glutamine, EAA, NEAA, NaH2PO4.H2O, MgCl2.6H2O glucose and media were purchased from Sigma-Aldrich (St. Louis, MO, USA).

#### Pretreatment solution (EG20)

Ethylene glycol (EG, Sigma) was diluted to 20% (v/v) in PBS-PVA.

#### Vitrification solution (EFS35)

Ethylene glycol was diluted to 35% (v/v) with FS solution to make EFS35. FS solution was PBS medium containing 30% (w/v) Ficoll 70 (average molecular weight 70,000; Sigma, USA) +0.5 M Sucrose.

#### Dilution solution

0.1% (w/v) PVA in PBS.

### Animal Studies

All experimental animal protocols were approved and performed in accordance with the requirements of the Institutional Animal Care and Use Committee at Chinese Academy of Agricultural Sciences.

### Blastocyst Production

Bovine ovaries were collected from the local abattoir (Xin Cheng Meat Co., Dachang Hui Autonomous County, Hebei Province) and transported to the laboratory within 2 hr. Cumulus-oocyte complexes (COCs) were aspirated from the follicles (2–8 mm in diameter). Three or more layers of compact cumulus cells were used for *in vitro* maturation.

#### In vitro maturation (IVM)

Groups of 50 COCs were washed three times with Dulbecco's phosphate buffered saline (PBS) (Invitrogen Corporation) containing 0.1% (wt/vol) polyvinyl alcohol (PVA) and then cultured in 4-well dishes (Nunclon, Roskilde, Denmark) in maturation medium at 38.5 °C and 5% CO_2_. IVM was performed for 24 hr in 700 μl of culture medium (M199, Gibco, Carlsbad, CA, USA) supplemented with 10 μg/ml follicle stimulating hormone (FSH, F8174, Sigma-Aldrich, St. Louis, MO, USA), 10 μg/ml luteinizing hormone (LH, L5269, Sigma-Aldrich, St. Louis, MO, USA), 10% (v/v) fetal bovine serum (FBS, Hyclone; Gibco BRL), and 10 μg/ml estradiol(E2, E2257, Sigma-Aldrich, St. Louis, MO, USA).

#### In vitro fertilization (IVF)

After maturation, the oocytes were washed three times with Brackett and Oliphant (BO) wash medium, aliquoted in groups of 15–20 and washed three times with BO fertilization medium consisting of 10 mM caffeine sodium benzoate and 0.5% fatty acid-free BSA, then transferred in a 50-μl drop of fertilization medium to a petri dish (Nunclon, Roskilde, Denmark) and placed under mineral oil at 5% CO_2_ in humidified air at 38.5 °C. Frozen semen was thawed at 37°C for 30 sec. The sperm were washed two times by centrifugation at 1800 rpm for 8 min in 3 ml of BO wash medium. Following the final wash, the motility and concentration of the sperm were determined. The pelleted sperm were re-suspended in BO wash medium to a volume of 1 ml. A 50-μl aliquot of the sperm suspension was added to each fertilization drop, giving a total concentration of 10×10^6^ spermatozoa/ml. The oocytes and sperm were incubated together at 5% CO_2_ in humidified air at 38.5°C for 8 hr before *in vitro* culturing.

#### In vitro culture (IVC)

A total of 15–20 zygotes were cultured in 60 μl of CR1aa+BSA (3 mg/ml, embryo culture tested fraction V, A-3311) medium that was supplemented with or without 10^−7^ M melatonin for 2 days and subsequently cultured in 60 μl of CR1aa+FBS (fetal bovine serum, 6% (v/v)) medium that was supplemented with or without 10^−7^ M melatonin. The concentration of 10^−7^ M melatonin was the optimal concentration repored in our previous research [35] from Day 3 until Day 8. Every 2 days, half of the medium was replaced. Cleavage rate was recorded at 48 h and blastocyst development recorded at Day 7 postinsemination. Six replicates were carried out.

### Vitrification and thawing of vitrified blastocyst

#### Vitrification

The room temperature for treatment of embryos was 25°C(±0.5). Embryos were suspended in a pretreatment solution (EG20) for 3 min and then pipetted into section of EFS35 in the straws to equilibrate for 30 sec. Then the straws were sealed and plunged into liquid nitrogen directly. Each straw took two blastocysts (Figure S1A, S1B).

#### Thawing

The room temperature was 25°C(±0.5). The straws were taken out from liquid nitrogen and plunged into water at 25°C(±0.5) for 10 sec. As soon as the crystallized solutions in the straw had melted, the straws were removed from the water and quickly wiped dry. The vitrification solution and the dilution solution were mixed in the straw as described in Figure S1C. When the embryos had been suspended in the mixture for 30 sec, the seals of the straw were moved and the embryos were expelled out of the straw into a dry culture dish. Embryos were then collected under a microscope and washed three times with PBS-PVA, then cultured in IVC medium in a CO_2_ incubator for 72 h.

### Quality assessment after thawing: mortality and hatching rates

Embryos, which had been collected post thawing were re-divided into melatonin-treated or control groups, each contains 10 to 15 embryos. They are cultured in *in vitro* under the condition mentioned above. Then they were examined at 12 h, 24 h, 36 h, 48 h, 60 h and 72 h after thawing. Mortality rate was defined as without re-expansion or development retarded blastocyst at the 12 h, 24 h, 36 h, 48 h, 60 h and 72 h after thawing, respectively. The hatching rate was also recorded.

### Determination of the relative abundance of the developmentally important gene transcripts in bovine blastocyst

Eight developmentally important genes (DNA methyltransferase 3a (*DNMT3A*), glucose transporters 3 (*SLC2A3*), Interferon_tau (*IFNT2*), occludin (*OCC*), tight junction protein1 (*TJP1*), Heat shock protein 1A (*HSPA1A*), aquaporin 3 (*AQP3*) and cadherin (*CDH1*)) were analyzed in hatched embryos that had previously been vitrified in melatonin treated and control group, respectively. The embryos were washed twice with D-PBS solution, and stored at −80°C until the RNA was extracted. The total RNA was extracted using TRIzol reagent (Invitrogen. Inc, California, USA), quantified by measuring the absorbance at 260 nm and stored at −80°C until use. The levels of relevant mRNAs were determined by quantitative RT-PCR using a One Step SYBR PrimeScript RT-PCR Kit (TaKaRa Bio. Inc., Tokyo, Japan) in a Light Cycler instrument (Roche Applied Science, Mannheim, Germany). The levels of accumulated fluorescence were analyzed using the second-derivative method after the melting-curve analysis was completed, and then the expression levels of the target genes were normalized to the expression level of β-actin in each sample. The primer pairs for the analyzed mRNAs are listed in Table 1.

**Table 1:** Primers used in this study.

Genes	Primer sequence(5′-3′)	Fragment Size(bp)	Sequence references and accession no.
*DNMT3A*	Forward:CCGTAGTGTCCAAGACCAATC	186	BC114063
	Reverse:GCTGAGTCAAATCCTCGTAAC		
* SLC2A3*	Forward:CCTGCTTTATTGTGGCTGAAC	165	NM_174603
	Reverse:CGAGGAACACAGTGAAGACGA		
* IFNT2*	Forward: CTCCAGCAGTGCCTCAACCT	158	NM_001015511
	Reverse:CCTCCCATGTCAGAGTCTTTCTC		
* OCC*	Forward: GTGTTGCCTCCACTCTTGCCT	177	BC133617.1
	Reverse: GGAATCCCTTTGCCGCTCT		
* TJP1*	Forward: GTCCTCTTCCTGCTTGACCTC	100	AJ313183
	Reverse: CACCCACATCGGATTCTACG		
* HSPA1A*	Forward: CGATGGTGCTGACCAAGATG	103	NM_174550
	Reverse: CGCTGCGAGTCGTTGAAGT		
* AQP3*	Forward: TGGCTTCAACTCTGGCTACG	236	NM_001079794
	Reverse: GCGACAACTTCACATTCTCCTC		
* CDH1*	Forward: CGTATCGGATTTGGAGGGAC	192	AY508164
	Reverse: CGAGGAACAAGAGCAGGGTG		
* β-actin*	Forward:TGACGTTGACATCCGTAAAGACC	117	NM_173979.3
	Reverse: GTGCTAGGAGCCAGGGCAG		

### Statistics Analysis

The data are expressed as mean ± SEM. The data were analyzed with univariate analysis of variance (ANOVA) followed by Duncan's test using SPSS 19.0 statistical software. The significant difference between treatments was set at P<0.05.

## Results

### Embryo Development

**Figure 1:**
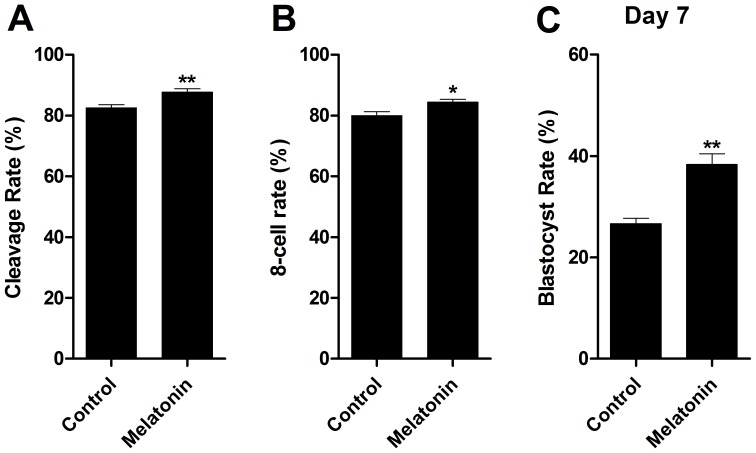
Effects of melatonin on the developments of *in vitro* maturation/fertilization bovine embryos. A: cleavage rate; B: 8-cell rate; C: blastocyst rate at Day 7 (*in vitro* fertilization = Day 0). Different superscripts in each column present statistical significant differences (P<0.05).

A total of 235 embryos were cultured in IVC medium to examine the potential effects of melatonin (10^−7^ M) on embryo development. The cleavage rate was significantly higher in melatonin treated samples (87.78±1.02%) than that in controls (82.50±1.12%, P<0.05) (Figure 1A). Similarly, the overall 8-cell embryo yield was also significantly higher in melatonin treated group (84.45±0.93%) than that in control group (80.00±1.29%, P<0.05) (Figure 1B). In addition, the kinetics of blastocyst appearance was affected by melatonin at Day 7, significantly more blastocysts were observed in melatonin-treated embryos (38.33±2.21%) than that in controls (26.67±1.05%, P<0.05) (Figure 1C).

### Cryotolerance

**Figure 2:**
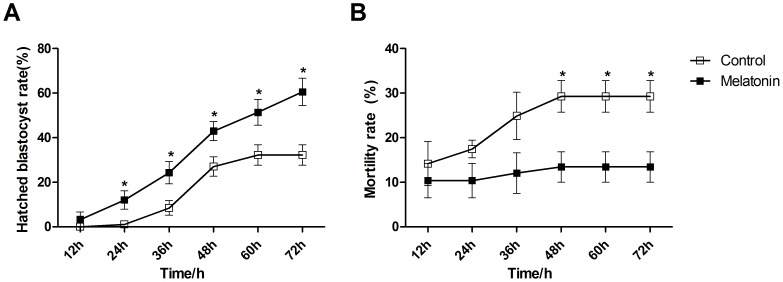
Effects of melatonin on blastocyst re-expansion and survival following vitrification and thawing. A: hatched blastocyst rate at different times after thawing. B: mortality rate at different times after thawing. Different superscripts indicate significant differences (P<0.05) between treatments at a given time point.

The ability of the blastocyst to withstand cryopreservation was used as an indicator of embryo quality. Embryos that had reached the stage of an expanding blastocyst (expanding blastocyst Day 7) were vitrified. A total of 54 blastocysts derived from melatonin-treated group and 65 blastocysts derived from control group were cultured in IVC medium to examine the potential cryotolerance after vitrification and thawing in this study. The experiment was repeated four times. The hatched blastocyst rate and mortality rate were examined from 12 h to 72 h after thawing. The hatched blastocyst rate did not show any significant difference between melatonin treated and control group at 12 h after thawing (P>0.05). However, the hatched blastocyst rates at 24 h, 36 h, 48 h, 60 h and 72 h after thawing were significantly higher (P<0.05) in melatonin (10^−7^ M) treated group (12.05±4.14%, 24.32±5.00%, 42.98±4.28%, 51.36±5.79% and 60.58±6.09%, respectively) than in the control (1.09±1.09%, 8.50±3.29%, 27.05±4.31%, 32.24±4.59% and 32.24±4.59%, respectively) (Figure 2A; Figure S2). It appeared that in melatonin treated group the hatched blastocyst rate continuously increased with time; however, in the control group this rate failed to further rise from 48 h to 72 h after thawing. In addition, blastocyst initial hatching time in control group was delayed compared to the melatonin treated group. The mortality rate in melatonin treated group showed a tendency of lower than that in control from 12 h to 36 h after thawing and this tendency reached statistically significance from 48 h to 72 h after thawing (Figure 2B; Figure S2). In addition, the mortality rate in melatonin treated group after thawing kept in low level during the process of blastocyst culture, in contrast, the mortality rate of thawed blastocyst in control group was continuously rising.

### Relative Transcript Abundance

**Figure 3:**
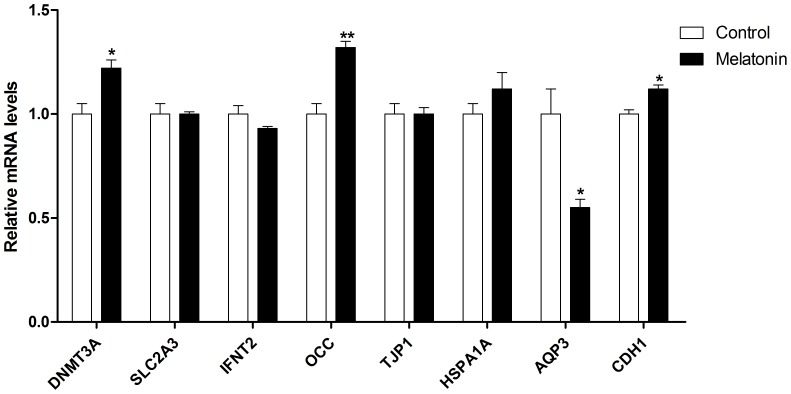
Effects of melatonin on the gene expression of developmentally important genes. Eight developmentally important genes (DNMT3A, SLC2A3, IFNT2, OCC, TJP1, HSPA1A, AQP3, CDH1) were detected in bovine blastosyst after thawing. The letters of a and b represent statistically significant differences (P<0.05).

A total of 44 blastocysts derived from melatonin-treated group and 46 blastocysts in control group were collected to examine the relative abundance of the developmentally important gene transcripts in bovine blastocyst. The experiment was repeated three times. At the molecular level, the gene expressions of DNA methyltransferase 3a (*DNMT3A*); Occludin (*OCC*); Cadherin (*CDH1*), were significantly higher in melatonin treated samples than that in controls (P<0.01–0.05) and in contrast, aquaporin 3 (*AQP3*), expression was significantly lower in melatonin treated group than that in control group (P<0.05) (Figure 3). Several other gene expressions including glucose transporters 3 (*SLC2A3*), interferon_tau (*IFNT2*), heat shock protein 1A (*HSPA1A*) and tight junction protein1 (*TJP1*) did not altered with melatonin treatment (P>0.05).

## Discussion

In order to better understand the complex nature of melatonin on *in vitro* produced embryo, its developmental competence (cleavage, 8-cell and blastocyst rates) and its cryotolerance with the relevant gene expression were investigated. It was reported that the embryo quality and viability is mainly affected by the culture system following IVF [13], [36]. Melatonin, as a potent free radical scavenger and antioxidant, is widely used in protection of *in vitro* cultured embryos. For example, melatonin added into culture medium increased the early mouse embryonic development [23]. The beneficial effects of melatonin on IVP embryo development included the enhancement of the blastocyst formation rate, mean cell number/blastocyst and the rate of hatch ability [28] and these benefits are observed in variety of species. For example, melatonin improved the quality and survival of ovine embryos in the *in vitro* and *in vivo* conditions [29], [37] and increased porcine embryo cleavage rates and blastocyst total cell numbers [26]. Under high oxygen concentration melatonin application also promoted *in vitro* produced bovine embryo development [25]. In addition, melatonin has been successfully used to increase the efficiency of blastocyst implantation in murine [32]. Here, we reported that melatonin accelerated the process of *in vitro* produced embryo development. This was indicated by more blastocysts being observed at Day 7 of culture in melatonin treated embryos than that of controls. This observation is similar to others [38], [39] in which they also reported that the *in vitro* culture systems influenced the kinetics of blastocyst development of bovine embryos. The evaluation or selection of bovine embryos for transfer or freezing is usually conducted at Day 7 of culture because at this time, normally developed embryos have reached, at least, the early blastocyst stage and in the most case a majority of them are at blastocyst stage and some at the expanded blastocyst stage [40]. If embryos developed to these stages delayed to Day 8 or even to Day 9 of culture they are considered to be poor quality and their utilities are limited [41].

*In vitro*-produced bovine embryos differ from those produced *in vivo* in many important respects [42]. These include retarded development and morphological appearance [2], [3], altered metabolism [4], higher thermal sensitivity [8], reduced expression of intercellular communicative devices [9] and, ultimately, in decreased pregnancy rates [10] following the *in vitro* culture. These differences contribute to the higher sensitivity to cryoinjury in the *in vitro* produced embryos [16]. An ability of an embryo to withstand freezing and thawing has been used as a useful indicator of its quality [13], [16], [43]. The different culture systems have profound effects on embryo freezability [17]. For example, if the *in vitro* produced zygotes were implanted into the sheep oviduct they have the greatest potential to survive freezing and thawing; however, if these zygotes were cultured with serum-supplemented media, such as synthetic oviduct fluid they exhibited low cryotolerance [44]. Here, for the first time, we observed that melatonin supplemented into IVC medium had a dramatic effect on blastocyst cryotolerance. This was indicated by significantly higher hatched blastocyst rate at 24 h, 36 h, 48 h, 60 h and 72 h after thawing in melatonin treated group than that in the controls (Figure 2). It was well documented that improved blastocyst quality significantly increased the pregnancy rate after transplantation, and resulted in more healthy offspring [45], [46].

It is well documented that *in vitro* culture conditions can also alter gene expression of embryo [12], [47], [48]. The analysis of mRNA expression may explain the observed differences in cryotolerance between *in vivo*- and *in vitro*-produced embryos [13], [16]. *DNMT3A* function as de novo methyltransferases that played important roles in normal development and disease [49] and it was also required for methylation of most imprinted loci in germ cells [50]. *OCC* is an integral membrane protein and localized at tight junctions [51]. it directly involves in the timing of blastocoel formation and, therefore, affects subsequent embryo development [52], [53]. *CDH1* is responsible for intercellular connectivity. Melatonin treatment resulted in significantly up-regulation of these three important genes which are closely related to the *in vitro* embryo development. Therefore, the quality improvement of embryo, at least, is partially attributed to the melatonin's effects on these genes up-regulation. *AQP3* is one of the transmembrane channel protein that functions as molecular water channels allowing water to flow rapidly across the membrane in the direction of osmotic gradients, mostly in epithelial tissues [54]. Previous study demonstrated that the expression of *AQP3* transcript was affected neither by the different culture conditions nor by the process of freezing of the blastocysts [33]. In the present study, melatonin significantly reduced the expression of *AQP3*. It was reported that decreased aquaporin expression leads to increased resistance to apoptosis [55]. Therefore, the effect of melatonin in protection IVP embryos against apoptosis may be through down-regulation of *AQP3*. *SLC2A3* is a very sensitive marker for suboptimal culture conditions and it is associated with poor quality of the embryos [56]. This gene was significantly up-regulated in the *in vitro* produced-embryos compared to their *in vivo* counterparts [57]. However, in this study, melatonin did not modify gene expression of *SLC2A3*. The relative quantification of other genes including *SLC2A3*, *IFNT2*, *HSPA1A* and *TJP1* were no significantly difference between groups with or without melatonin treatment. It appeared that these genes did not participate in the activities of melatonin on *in vitro* embryo development.

In conclusion, our results show that melatonin improves the kinetics of *in vitro* produced-embryo development and the quality of the resulting blastocysts and increases cryotolerance of blastocysts. The mechanisms are multiples. One of the most important mechanisms is to modify the expressions of several embryo-developmentally important genes. These include up-regulating *DNMT3A*, *OCC*, *CDH1* and down-regulating *AQP3*.

## Supporting Information

Figure S1 **Vitrification and thawing protocols in the present study.** A. vitrification protocol; B: configuration of the straw. Approximately 7.5 cm of a dilution solution (PBS-PVA), a small volume of EFS35, and approximately 1 cm of EFS35 (take embryos) were aspirated into the straw one by one and each chamber was separated by small air bubbles. Then the embryos were pipetted into the section of EFS35 in the straw. Another volume of the dilution solution was aspirated and then the open end of the straw was sealed with PVA powder; C: solutions in the straw move from one end to another and the EFS35 was mixed with PBS-PVA solutions little by little.
(TIF)

Figure S2 **Effects of melatonin on the development of bovine embryos after vitrification and thawing.** The red arrows represent dead blastocyst (defined as without re-expansion or development retarded blastocyst) and blue arrows represent hatched blastocyst (hatching is a phenomenon seen with embryos developing outside the body. Under natural conditions inside the body, the zona is believed to degenerate and disappear after the embryo reaches the uterine cavity and prepares to implant). Blastocysts were produced in IVC medium supplemented with or without 10^−7^ M melatonin, following vitrification and thawing at Day 7 of embryo culture, then cultured in IVC medium in a CO_2_ incubator for 72 h. melatonin (10^−7^ M) significantly increased the hatched blastocyst rate from 24 h to 72 h and decreased the mortality rate from 48 h to 72 h after thawing.
(TIF)
