# Supplementary figures and images for: Melatonin Improves the Quality of In Vitro Produced (IVP) Bovine Embryos: Implications for Blastocyst Development, Cryotolerance, and Modifications of Relevant Gene Expression
Source: PLoS One. 2014 Apr 2;9(4):e93641. doi: 10.1371/journal.pone.0093641 (PMC3973586; doi:10.1371/journal.pone.0093641)

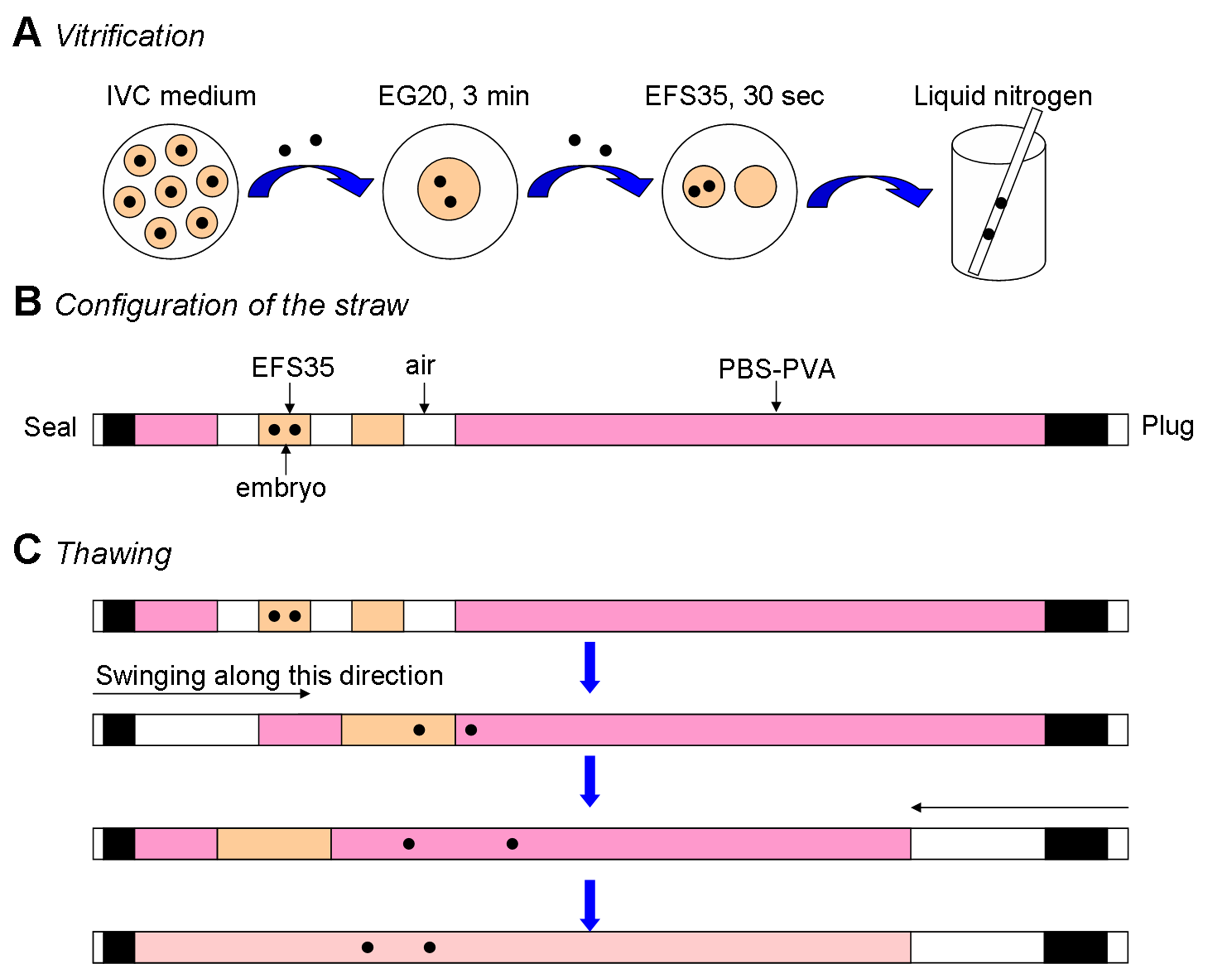

Supplement: Figure S1 — Vitrification and thawing protocols in the present study. A. vitrification protocol; B: configuration of the straw. Approximately 7.5 cm of a dilution solution (PBS-PVA), a small volume of EFS35, and approximately 1 cm of EFS35 (take embryos) were aspirated into the straw one by one and each chamber was separated by small air bubbles. Then the embryos were pipetted into the section of EFS35 in the straw. Another volume of the dilution solution was aspirated and then the open end of the straw was sealed with PVA powder; C: solutions in the straw move from one end to another and the EFS35 was mixed with PBS-PVA solutions little by little. (TIF) [file pone.0093641.s001.tif]

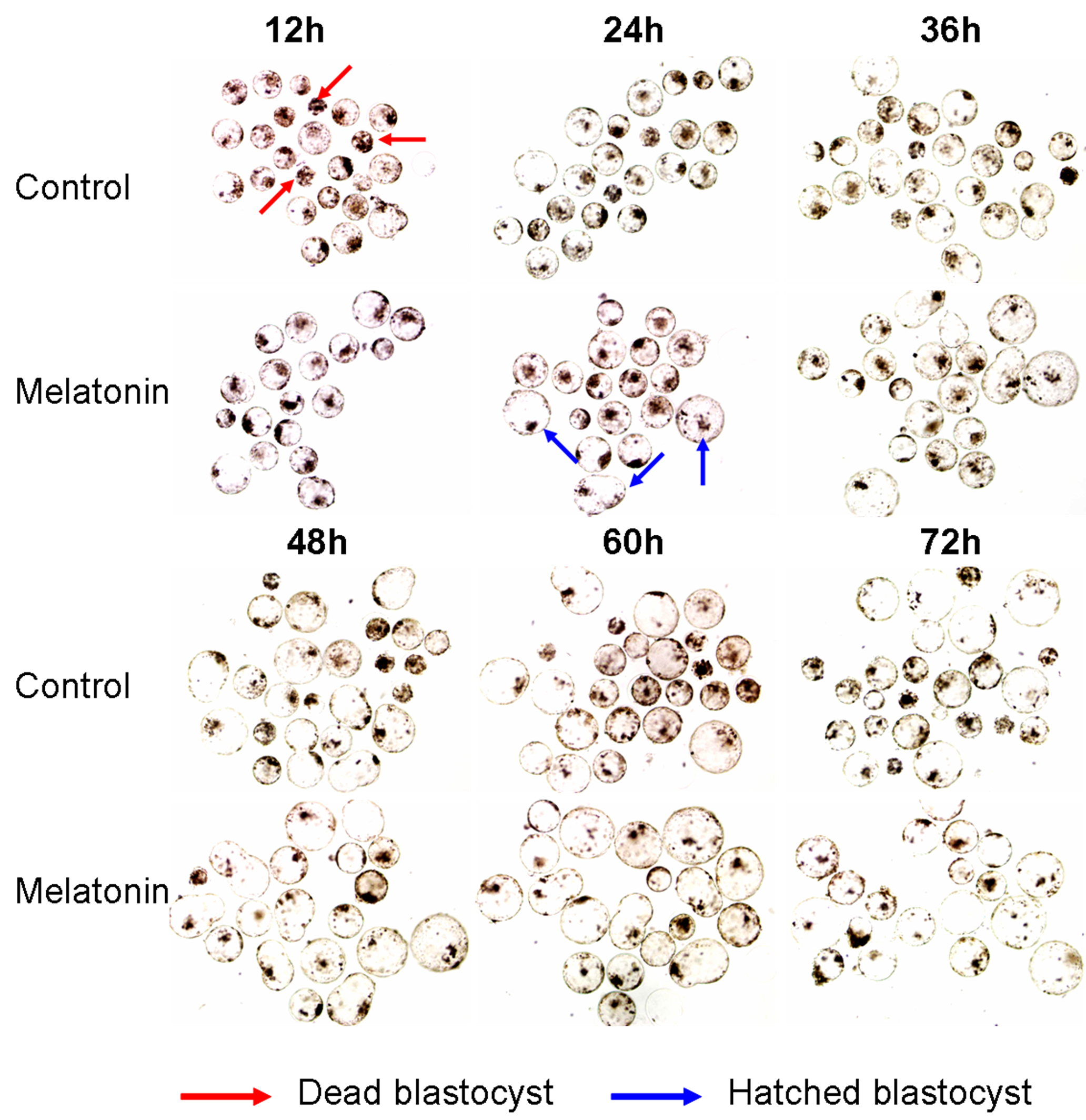

Supplement: Figure S2 — Effects of melatonin on the development of bovine embryos after vitrification and thawing. The red arrows represent dead blastocyst (defined as without re-expansion or development retarded blastocyst) and blue arrows represent hatched blastocyst (hatching is a phenomenon seen with embryos developing outside the body. Under natural conditions inside the body, the zona is believed to degenerate and disappear after the embryo reaches the uterine cavity and prepares to implant). Blastocysts were produced in IVC medium supplemented with or without 10−7 M melatonin, following vitrification and thawing at Day 7 of embryo culture, then cultured in IVC medium in a CO2 incubator for 72 h. melatonin (10−7 M) significantly increased the hatched blastocyst rate from 24 h to 72 h and decreased the mortality rate from 48 h to 72 h after thawing. (TIF) [file pone.0093641.s002.tif]
